# ‘Cryptic’ exons reveal some of their secrets

**DOI:** 10.7554/eLife.00476

**Published:** 2013-01-22

**Authors:** John A Calarco

**Affiliations:** 1 **John A Calarco** is at the FAS Center for Systems Biology, Harvard University, Cambridge, United Statesjcalarco@fas.harvard.edu

**Keywords:** HITS-CLIP, Nonsense mediated decay, alternative splicing, RNA regulation, epilepsy, neuronal biology, Mouse

## Abstract

By regulating the inclusion of ‘cryptic’ exons in messenger RNA in nerve cells, NOVA proteins are able to influence the abundance of the corresponding proteins.

**Related research article** Eom T, Zhang C, Wang H, Lay K, Fak J, Noebels JL, Darnell RB. 2013. NOVA-dependent regulation of cryptic NMD exons controls synaptic protein levels after seizure. *eLife* **2**:e00178. doi: 10.7554/elife.00178

**Image** NOVA proteins predominantly bind RNAs in intronic regions (red) as opposed to 3’ untranslated regions (blue)

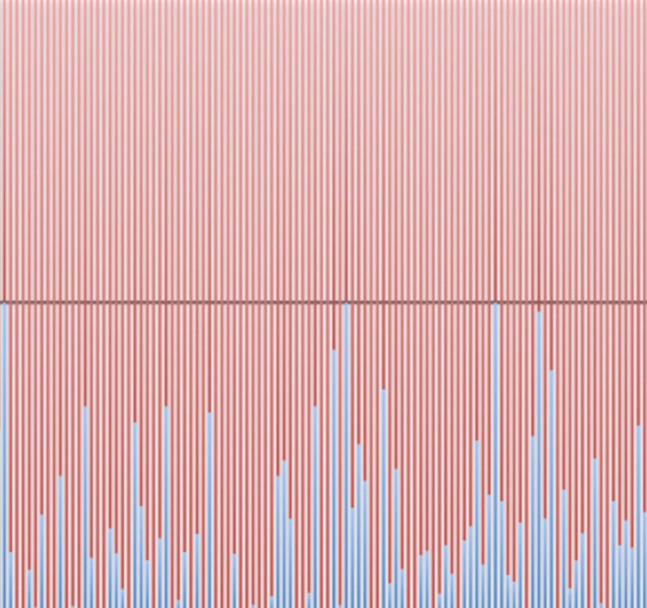

When the DNA in the genes of a eukaryote has been transcribed into RNA, sequences of bases known as introns are then removed from the RNA transcript, and the remaining sequences, which are called exons, are spliced back together to produce messenger RNA. Most of the time this mRNA is then translated into a string of amino acids, which subsequently folds to form a protein. It has been known for some time that certain exons can be included or excluded when splicing together the mature mRNA. This form of splicing, which is known as alternative splicing, is generally considered to be a mechanism for allowing a single gene to code for two or more proteins (Nilsen and Graveley, 2010).

However, it is also known that alternative splicing can contribute to mechanisms that are used to control the abundances of certain proteins: in particular, mRNA degradation pathways in cells can target mRNA that has been spliced in a certain way. Now, writing in *eLife*, Taesun Eom and Robert Darnell of Rockefeller University, and co-workers at Rockefeller and Baylor College of Medicine, shed new light on these processes by identifying a previously unknown class of exons in RNA transcripts produced in neuronal cells (Eom et al., 2013). These ‘cryptic’ exons can be regulated by changes in neuronal activity, and might also help the brain to recover from seizures.

Over the years Darnell and co-workers have investigated the roles of RNA binding proteins in the nervous system. In particular, they have studied RNA binding proteins known as NOVA proteins and shown that they have a critical role in the cell nucleus, where they help to regulate alternative splicing (Ule et al., 2005). These NOVA proteins can also shuttle from the nucleus to the cytoplasm, and it is thought that they can modulate the abundances of proteins in neuronal cells by helping to localize RNA to dendrites (Racca et al., 2010).

To explore the differences between the nuclear and cytoplasmic roles of NOVA, Eom and colleagues separated brain tissue from mice into nuclear and cytoplasmic fractions, and employed a technique called HITS-CLIP (Licatalosi et al., 2008) to study the binding between the NOVA proteins and the RNA in these two fractions. They found that in the nucleus the NOVA proteins bind primarily to intronic regions of the RNA transcript: however, in the cytoplasm they bind mostly to 3′ untranslated regions of the transcripts. The Rockefeller-Baylor team then studied mice in which the genes for NOVA proteins had been knocked out, and found hundreds of RNA transcripts that were present at lower levels in these animals than in wild-type mice: they also found a smaller number of RNA transcripts that were present at higher levels in the knockout mice. Moreover, they found that NOVA proteins predominantly bind to these RNA transcripts in the nucleus, which was initially surprising because RNA binding proteins are generally thought to regulate mRNA levels by binding to the untranslated regions of RNA transcripts in the cytoplasm.

These results led Eom and co-workers to explore the intronic regions that the NOVA proteins bind to: they wanted to see if these regions contained exons that could be included in mRNA, and this is indeed what they found. In general these cryptic exons were found at low levels in the mature mRNA of wild-type mice, and at much higher levels in knockout mice (although a small number of RNA transcripts contain high numbers of cryptic exons in wild-type mice and low numbers in knockout mice). When included in RNA transcripts, these cryptic exons introduced premature translation stop codons into the mRNA. Instead of allowing full-length proteins to be produced, these codons trigger an mRNA degradation pathway called nonsense-mediated mRNA decay (Schoenberg and Maquat, 2012). The end result, therefore, is that cryptic exons can be used to influence the levels of proteins and mRNA in the cell (Figure 1).

**Figure 1.**
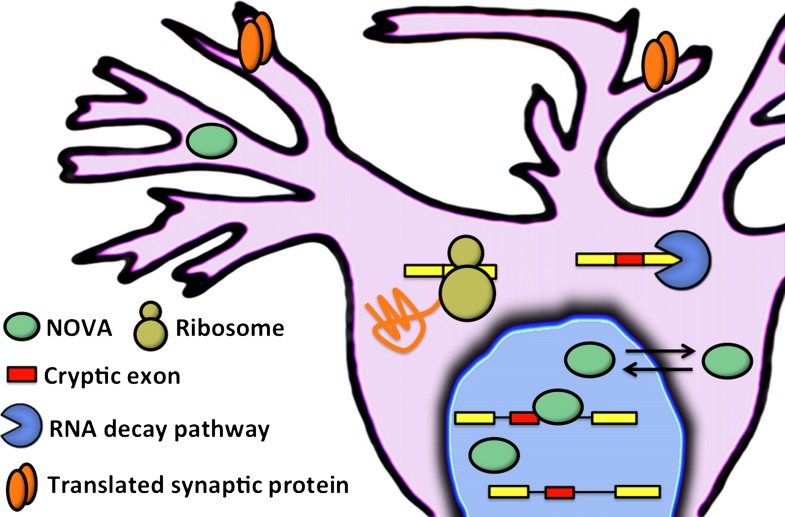
Eom and co-workers have shown that NOVA proteins (green ellipses) regulate the inclusion of cryptic exons (red boxes) in messenger RNA (mRNA) in the nucleus of neuronal cells. In the cytoplasm, mRNA that contains cryptic exons is targeted by RNA decay pathways (shown here by the blue pac-man), while mRNA that does not contain cryptic exons is translated by the ribosome (yellow) to produce synaptic proteins (orange). Neuronal activity can cause the NOVA proteins to shuttle from the nucleus to the cytoplasm: this changes the proportion of cryptic exons that are included in the mRNA, and therefore alters the abundance of the corresponding proteins.

In further experiments designed to explore the physiological relevance of this novel layer of NOVA-mediated gene regulation, the Rockefeller-Baylor team found that treating mice with a compound called pilocarpine, which modulates neuronal activity and induces seizures in animals, led to increased inclusion of certain cryptic exons. Intriguingly, in some regions of the brain, NOVA proteins move from the nucleus to the cytoplasm after treatment with pilocarpine. Moreover, mice that have lost one copy of the gene that codes for a specific NOVA protein (called NOVA 2) display spontaneous epileptic episodes. These results suggest that there is a connection between neuronal activity-dependent redistribution of NOVA proteins in the cell, the regulation of transcript levels by alternative splicing coupled to nonsense-mediated decay, and a requirement for NOVA proteins in maintaining balance in neuronal activity, potentially after excitatory stress.

This work is exciting for several reasons. First, the identification of a network of neuronal transcripts that is regulated by the coordinated action of alternative splicing and nonsense-mediated decay is a significant advance. It is well known that the coupling of these two gene regulatory layers has an important role in regulating the abundance of splicing factors, RNA binding proteins, and core components of the splicing machinery (Lareau et al., 2007; Saltzman et al., 2008). There have also been examples of individual splicing events in genes with important roles in neuronal development or function that are subject to this type of regulation (Boutz et al., 2007; Zheng et al., 2012). However, the set of transcripts identified in this latest study are all regulated in a coordinated manner by NOVA proteins, indicating that they likely contribute to aspects of neuronal biology as a module. In agreement with this idea, Eom and co-workers find that genes with NOVA-dependent cryptic exons often encode proteins enriched in similar functions at the synapse. A future goal will be to understand how this network of genes helps protect neurons from stress induced by neuronal activity. The results of such an analysis could yield new insights into and treatments for epilepsy.

Second, this work has also unmasked an important hidden layer of biology. The identification of cryptic exons regulated by NOVA would not have occurred without the use of HITS-CLIP and knockout mice because these exons are only present at very low levels (or are completely absent) in the transcripts of wild-type animals under normal behavioural conditions. These latest findings highlight the importance of performing more experiments on context-specific gene expression in dynamically regulated cells such as neurons. Future research involving RNA binding proteins other than NOVA proteins is sure to capture additional target transcripts subject to dynamic gene regulation in neurons. Indeed, several recent studies have demonstrated that this will likely be the case (Iijima et al., 2011; Yap et al., 2012), and provide an indication that there is much more uncharted RNA biology waiting to be decrypted.

## References

[bib1] Boutz PL Stoilov P Li Q Lin CH Chawla G Ostrow K 2007 A post-transcriptional regulatory switch in polypyrimidine tract-binding proteins reprograms alternative splicing in developing neurons. Genes Dev 21:1636–52 doi: 10.1101/gad.1558107 17606642 PMC1899473

[bib2] Eom T Zhang C Wang H Lay K Fak J Noebels JL 2013 NOVA-dependent regulation of cryptic NMD exons controls synaptic protein levels after seizure. eLife 2:e00178 doi: 10.7554/elife.00178 PMC3552424 23359859

[bib3] Iijima T Wu K Witte H Hanno-Iijima Y Glatter T Richard S 2011 SAM68 regulates neuronal activity-dependent alternative splicing of neurexin-1. Cell 147:1601–14 doi: 10.1016/j.cell.2011.11.028 22196734 PMC3246220

[bib4] Lareau LF Brooks AN Soergel DA Meng Q Brenner SE 2007 The coupling of alternative splicing and nonsense-mediated mRNA decay. Adv Exp Med Biol 623:190–211 18380348 10.1007/978-0-387-77374-2_12

[bib5] Licatalosi DD Mele A Fak JJ Ule J Kayikci M Chi SW 2008 HITS-CLIP yields genome-wide insights into brain alternative RNA processing. Nature 456:464–69 doi: 10.1038/nature07488 18978773 PMC2597294

[bib6] Nilsen TW Graveley BR 2010 Expansion of the eukaryotic proteome by alternative splicing. Nature 463:457–63 doi: 10.1038/nature08909 20110989 PMC3443858

[bib7] Racca C Gardiol A Eom T Ule J Triller A Darnell RB 2010 The neuronal splicing factor Nova co-localizes with target RNAs in the dendrite. Front Neural Circuits 4:5 doi: 10.3389/neuro.04.005.2010 20407637 PMC2856630

[bib8] Saltzman AL Kim YK Pan Q Fagnani MM Maquat LE Blencowe BJ 2008 Regulation of multiple core spliceosomal proteins by alternative splicing-coupled nonsense-mediated mRNA decay. Mol Cell Biol 28:4320–30 doi: 10.1128/MCB.00361-08 18443041 PMC2447145

[bib9] Schoenberg DR Maquat LE 2012 Regulation of cytoplasmic mRNA decay. Nat Rev Genet 13:246–59 doi: 10.1038/nrg3160 22392217 PMC3351101

[bib10] Ule J Ule A Spencer J Williams A Hu JS Cline M 2005 Nova regulates brain-specific splicing to shape the synapse. Nat Genet 37:844–52 doi: 10.1038/ng1610 16041372

[bib11] Yap K Lim ZQ Khandelia P Friedman B Makeyev EV 2012 Coordinated regulation of neuronal mRNA steady-state levels through developmentally controlled intron retention. Genes Dev 26:1209–23 doi: 10.1101/gad.188037.112 22661231 PMC3371409

[bib12] Zheng S Gray EE Chawla G Porse BT O’Dell TJ Black DL 2012 PSD-95 is post-transcriptionally repressed during early neural development by PTBP1 and PTBP2. Nat Neurosci 15:381–8 doi: 10.1038/nn.3026 22246437 PMC3288398

